# Surfactin-Producing *Bacillus velezensis* A1 Inhibits Lactic Acid Bacteria in *Jiang*-Flavor *Baijiu* Fermentation

**DOI:** 10.3390/foods15071140

**Published:** 2026-03-26

**Authors:** Xinyue Li, Menglin She, Lingfeng Yi, Guanyu Zhou, Yafei Lian, Chong Yang, Yaokang Wu, Yanfeng Liu, Ying Han, Jianghua Li

**Affiliations:** 1 Key Laboratory of Carbohydrate Chemistry and Biotechnology, School of Biotechnology, Ministry of Education, Jiangnan University, Wuxi 214122, China; 6230201021@stu.jiangnan.edu.cn (X.L.); 6220201051@stu.jiangnan.edu.cn (L.Y.); 6240210024@stu.jiangnan.edu.cn (Y.L.); yaokangwu@jiangnan.edu.cn (Y.W.); yanfengliu@jiangnan.edu.cn (Y.L.); 2 Science Center for Future Foods, Jiangnan University, Wuxi 214122, China; 3 Kweichow Moutai Co., Ltd., Maotai Town, Renhuai 564501, China; 201911110711017@stu.hubu.edu.cn (M.S.); 13481391969@163.com (G.Z.); yc82300@163.com (C.Y.); 4 Guizhou Key Laboratory of Microbial Resources Exploration in Fermentation Industry, Kweichow Moutai Group, Zunyi 564501, China

**Keywords:** high-temperature *Daqu*, microbial community, fermented food, antagonistic mechanism, microbiome, *Bacillus velezensis*, whole genome sequencing

## Abstract

Lactic Acid Bacteria contribute to heightened acidity in the fermentation process of *Jiang*-flavor *Baijiu* due to their production of lactic acid. High-temperature *Daqu* may act as a reservoir for beneficial microorganisms and antimicrobial compounds. In this study, we utilized 16S rRNA and ITS amplicon sequencing to identify microbial taxa in high-temperature *Daqu* that inhibit the primary lactic acid bacterium involved in *Jiang*-flavor *Baijiu* fermentation, *Acetilactobacillus jinshanensis*, followed by the selection of antagonistic strains. The strain exhibiting the strongest antagonistic activity was identified as *Bacillus velezensis* based on whole-genome sequencing. Genome analysis revealed 12 secondary metabolite biosynthetic gene clusters, from which one lipopeptide was identified. This lipopeptide was demonstrated to antagonize *A. jinshanensis* AJS1 by disrupting the cell membrane and inducing leakage of intracellular contents. Collectively, strain A1 and its secondary metabolites exhibit considerable promise as antagonistic agents to mitigate acidity increases triggered by *A. jinshanensis* AJS1 during the fermentation of *Jiang*-flavor *Baijiu*.

## 1. Introduction

During the fermentation of *Jiang*-flavor *Baijiu*, abnormal increases in acidity significantly inhibit the growth of yeast [1]. When the medium pH falls below the pKa of lactic acid (3.86), large amounts of undissociated lactic acid enter the cytoplasm, causing intracellular pH to rapidly decrease from 6.28 to 3.48. Consequently, the specific growth rate of yeast decreases from 0.260 h^−1^ to 0.099 h^−1^, and ethanol production is severely impaired [2]. Elevated acidity also intensifies sour and astringent sensations in *Jiang*-flavor *Baijiu*, leading to quality deterioration and reduced drinkability [3]. In the multi-round solid-state fermentation system of *Jiang*-flavor *Baijiu*, lactic acid constitutes the major fraction of organic acids, and its abnormal accumulation is the primary driver of increased acidity [4,5]. During the *Zaosha* stage, lactic acid content in fermented grains increases from 0.3% (*w*/*v*) to 1.3%, accounting for over 70% of total acids [6]. Therefore, regulating lactic acid levels during fermentation is critical for maintaining *Jiang*-flavor *Baijiu* quality.

Current strategies for acidity reduction in fermented foods include physical, chemical, process-based, and biological approaches. Physical methods include steam stripping and adsorption. Steam stripping can remove volatile acids but are ineffective against non-volatile lactic acid [7]. Adsorption requires specialized equipment and introduces risks related to adsorbent separation and residues, which may compromise liquor quality [8]. Chemical neutralization introduces exogenous reagents that can negatively affect flavor [9]. Moreover, physical and chemical methods only address apparent physicochemical properties and fail to resolve acidity at its microbial source, limiting sustainability. Process optimization can reduce acid production by acidogenic microorganisms [10]; however, changes in fermentation parameters (e.g., temperature) significantly alter microbial communities and flavor profiles [11,12,13]. Biological approaches, which rely on microbial interactions to control lactic acid producers, have shown promise. For example, the introduction of lactic acid–degrading strains can reduce lactic acid levels without affecting characteristic flavors [14], and *Bacillus licheniformis* isolated from high-temperature *Daqu* has been shown to antagonize *Lactobacillus panis* [15]. Although engineered strains can reduce acidity [16], their safety and stability are difficult to guarantee in the complex production system of *Jiang*-flavor *Baijiu*. In contrast, indigenous microorganisms from the fermentation ecosystem are more controllable.

Metagenomic and metatranscriptomic analyses have demonstrated that *A. jinshanensis* rapidly becomes the dominant species during the second to third fermentation rounds, reaching a relative abundance of up to 92%. Correlation analyses further confirmed this species as the key driver of excessive lactic acid production and abnormal acidity increases [17]. Therefore, exploiting indigenous antagonistic microorganisms to inhibit *A. jinshanensis* represents a promising and sustainable strategy for acidity control. High-temperature *Daqu* serves as a critical carrier of microorganisms and metabolites in *Jiang*-flavor *Baijiu* fermentation and has strong potential as a source of antagonistic microbes and antimicrobial compounds [18,19,20]. Dominant genera in high-temperature *Daqu*, including *Bacillus*, *Saccharopolyspora*, *Kroppenstedtia*, and *Pichia*, are known to produce diverse antimicrobial secondary metabolites such as lipopeptides, lantipeptides, and volatile organic compounds [21,22,23,24,25]. Compared with traditional *Daqu*, fortified *Daqu* inoculated with *Bacillus subtilis* exhibits reduced abundances of *Weissella* and *Lactobacillus* [26]. However, the inhibitory effects of high-temperature *Daqu* on *A. jinshanensis* remain largely unexplored.

Amplicon and genome sequencing technologies provide powerful tools for identifying inhibitory factors in high-temperature *Daqu*, enabling the discovery of antagonistic microorganisms and antimicrobial biosynthetic gene clusters [27,28]. In this study, we integrated 16S rRNA and ITS amplicon sequencing, whole-genome sequencing of antagonistic strains, and antimicrobial compound identification to systematically investigate microorganisms and metabolites in high-temperature *Daqu* that inhibit *A. jinshanensis* AJS1. This work provides a solid foundation for biological acidity control during *Jiang*-flavor *Baijiu* fermentation.

## 2. Materials and Methods

### 2.1. Strains and Culture Media

The *Bacillus* strains used in this study were isolated from high-temperature *Daqu* as previously described [15], including 33 strains: *B. licheniformis* L1–L20, *Bacillus sonorensis* S1–S5, *Bacillus cereus* BC, *Bacillus velezensis* A1–A2, S6, V2, V4 and V5, and *Bacillus amyloliquefaciens* V3 (Table S1). *A. jinshanensis* AJS1 was obtained from the Moutai microbial bank (Renhuai City, Guizhou Province, China). This strain was initially isolated as L214 from pit fermentation grains using the plate dilution method and was identified through whole-genome sequencing, as previously described [17]. For clarity in this study, we have designated this strain as AJS1. *A. jinshanensis* AJS1 was cultivated in MRS-jinshanensis medium (MJS), which was formulated based on standard MRS medium supplemented with 350 mL/L fermented-grain extract, 15 g/L sodium acetate, 1 g/L L-cysteine, 10 mg/L FeSO_4_·7H_2_O, 10 g/L acetic acid, and 20 g/L lactic acid. All *Bacillus* strains were activated in LB medium and fermented in Landy medium. The composition of Landy medium was as follows: glucose (20 g/L), sodium L-glutamate (5 g/L), MgSO_4_ (0.5 g/L), KCl (0.5 g/L), KH_2_PO_4_ (1 g/L), FeSO_4_ (0.15 mg/L), MnSO_4_ (5 mg/L), and CuSO_4_ (0.16 mg/L).

### 2.2. Screening of High-Temperature Daqu and Sample Selection

A total of 934 high-temperature *Daqu* samples were collected and tested from a *Jiang*-flavor *Baijiu* distillery in Renhuai, Guizhou Province. *Daqu* extracts were prepared as follows: 4 g of *Daqu* powder was mixed with 8 mL of ultrapure water, vortexed at 2000 rpm for 10 min, and centrifuged at 10,000 rpm for 2 min. The supernatant was filtered through a 0.22 μm membrane (Zhejiang ALWSCI Technologies Co., Ltd., Shaoxing, China) to obtain the *Daqu* extract. *A. jinshanensis* AJS1 was cultivated in 96-well plates. Each 200 μL reaction system consisted of 160 μL MJS medium, 20 μL *A. jinshanensis* AJS1 seed culture, and 20 μL *Daqu* extract or MJS medium (control). Each *Daqu* sample was tested in triplicate. The initial OD_600_ was measured using BioTek microplate reader (Agilent Technologies, Santa Clara, CA, USA). Plates were incubated anaerobically at 30 °C for 3 days, after which OD_600_ was measured again. The OD_600_ increment before and after incubation was calculated. The relative growth rate (RGR) was defined as:

(1) RGR= (OD increment of Daqu extract−OD increment of control)/(OD increment of control)×100%

A negative RGR indicated inhibition of *A. jinshanensis* AJS1 growth.

Based on their effects on *A. jinshanensis* AJS1 growth, high-temperature *Daqu* samples were classified into four groups: inhibitory (I), low promoting (LP), moderately promoting (MP), and highly promoting (HP), with RGR ranges of <0, 0–60%, 60–120%, and >120%, respectively. Six samples were randomly selected from each group, homogenized, and stored at −20 °C for subsequent analyses.

### 2.3. 16S rRNA and ITS Amplicon Sequencing Analysis

16S rRNA and ITS amplicon sequencing was performed by Genesky Biotechnologies Inc. (Shanghai, China). Total genomic DNA was extracted using the FastDNA^®^ SPIN Kit for Soil (MP Biomedicals, Santa Ana, CA, USA) following the manufacturer’s instructions. DNA integrity was assessed by agarose gel electrophoresis, and DNA concentration and purity were determined using a NanoDrop 2000 (Thermo Fisher Scientific, Waltham, MA, USA) and Qubit 3.0 Spectrophotometer (Thermo Fisher Scientific).

The V3–V4 hypervariable regions of the bacterial 16S rRNA gene were amplified using primers 341F (5′-CCTACGGGNGGCWGCAG-3′) and 805R (5′-GACTACHVGGGTATCTAATCC-3′). The fungal ITS2 region was amplified using primers ITS3 (5′-GCATCGATGAAGAACGCAGC-3′) and ITS4 (5′-TCCTCCGCTTATTGATATGC-3′). Sequencing was conducted on an Illumina NovaSeq 6000 platform (Illumina, San Diego, CA, USA). Raw reads were processed using the QIIME 2 pipeline [29]. Adapter and primer sequences were removed using the cutadapt plugin, and quality control and amplicon sequence variant (ASV) inference were performed using DADA2 [30]. Taxonomic assignment of 16S rRNA ASVs was conducted using a pretrained Naive Bayes classifier against the SILVA 138.2 database (confidence threshold 0.7). ITS ASVs were annotated using the UNITE database (version 10.0) with a confidence threshold of 0.6.

### 2.4. Selection of Antagonistic Bacillus Strains and Growth Characterization

*Bacillus* strains were activated in LB medium and inoculated into Landy medium at an initial OD_600_ of 0.1, and cultures were fermented at 37 °C and 220 rpm for 48 h. Fermentation broths were centrifuged at 12,000 rpm for 10 min, and supernatants were collected. During fermentation, samples were collected every 12 h to determine biomass (OD_600_) and glucose consumption from 12 to 72 h. Residual glucose was measured using a biosensor analyzer (Shenzhen Sieman Technology Co., Ltd., Shenzhen, China), and OD_600_ was measured using a spectrophotometer (Agilent Technologies, Santa Clara, CA, USA). Phylogenetic tree based on *gyrA* was conducted in MEGA11 using the Neighbor-Joining method.

*A. jinshanensis* AJS1 was activated in MJS medium and inoculated into MJS agar plates at an initial OD_600_ of 0.05. Wells with a diameter of 6 mm were punched into the agar, and 10 μL of fermentation supernatant or control solution (1 mg/mL ampicillin) was added to each well. Plates were incubated anaerobically for 2 days, and inhibition zone diameters were measured using a digital caliper.

The relative inhibition rate was calculated as shown below, where the blank diameter corresponded to the agar punch diameter:

(2) $$Specific\;inhibition\;rate=\frac{\left(inhibition\;zone\;diameter\;of\;antagonistic\;supernatant-blank\;diameter\right)}{\left(inhibition\;zone\;diameter\;of\;1\;mg/mL\;ampicillin-blank\;diameter\right)}\times 100\%$$

### 2.5. Whole-Genome Sequencing, Annotation, and Identification of B. velezensis A1

Whole-genome sequencing of strain A1 was performed by Geneseq Biotechnology Company (Nanjing, China) using the Nanopore PromrthION48 platform/(Pacific Biosciences platform) and the Illumina Novaseq platform (Illumina, San Diego, CA, USA). Filtered reads were assembled using Flye [31] and Unicycler [32]. Assemblies were integrated and polished using Pilon [33] to generate the final genome sequence.

Gene prediction was conducted using GeneMarkS v4.32 [34]. tRNA and rRNA genes were identified using tRNAscan-SE [35] and Barrnap (v0.9), respectively. Functional annotation was performed using BLAST (v2.13.0) searches against the NR, GO, KEGG, and COG databases. Carbohydrate-active enzymes were predicted using the CAZy database [36]. Secondary metabolite biosynthetic gene clusters were identified using antiSMASH. Genome visualization was performed using Circos (v0.64).

Based on the assembled chromosome sequence, fastANI [37] was used to calculate average nucleotide identity (ANI) values against 20 closely related species. Phylogenomic analysis was performed using UBCG [38] based on 92 conserved single-copy core genes (Table S2). Comparative genome ring plots for the top three ANI-matched genomes were generated using BRIG [39].

### 2.6. Preparation of Crude Antimicrobial Lipopeptides

Fermentation broth was prepared following the antagonistic strain selection procedure. Crude lipopeptides were preliminarily isolated using an acid precipitation–methanol extraction method with subsequent condition optimization [40]. Briefly, fermentation supernatants were centrifuged at 10,000 rpm for 20 min, and the pH was adjusted stepwise to 7, 6, 5, 4, 3, and 2 using 6 M HCl. After incubation at 4 °C for 1 h to allow precipitation, samples were centrifuged again at 10,000 rpm for 20 min. The precipitates were extracted twice with methanol, followed by centrifugation at 10,000 rpm for 20 min. The combined supernatants were concentrated by centrifugation, and the optimal extraction pH was determined based on the relative inhibition rate of the re-dissolved crude lipopeptides.

### 2.7. Lipopeptide Identification by Thin-Layer Chromatography and Mass Spectrometry

Crude lipopeptides were spotted onto high-performance G-type silica gel thin-layer chromatography (TLC) plates (Labshark, Changde, China). Plates were developed using chloroform/methanol/water (65:25:5, *v*/*v*/*v*) as the mobile phase. Lipid moieties were visualized by spraying with distilled water, while free amino groups were detected using 0.2% ninhydrin solution followed by heating at 110 °C for 5 min [41].

Lipopeptide bands detected by TLC were recovered by preparative TLC and subjected to molecular weight determination using a Bruker Daltonics ultrafleXtreme MALDI-TOF mass spectrometer (Bruker Daltonics, Billerica, MA, USA) with 2-cyano-4-hydroxycinnamic acid as the matrix. The instrument was equipped with a Smartbeam II nitrogen laser (337 nm) (Bruker, Karlsruhe, Germany) and operated at an acceleration voltage of 25 kV, with an m/z scanning range of 500–2000.

Further structural characterization was performed using LC-MS/MS. Liquid chromatography was conducted on an Easy-nLC 1200 nano-flow system (Thermo Fisher Scientific, Waltham, MA, USA) equipped with a C18 reverse-phase column (Acclaim PepMap RSLC, 75 μm × 25 cm, 2 μm, 100 Å). Lipopeptides were eluted using 100% solvent B (80% acetonitrile containing 0.1% formic acid) for 30 min under isocratic conditions. Mass spectrometry was performed on an Orbitrap Fusion Lumos mass spectrometer (Thermo Fisher Scientific, Waltham, MA, USA) equipped with a Nano Flex ion source. The spray voltage was set to 1.9 kV, and the ion transfer tube temperature was maintained at 275 °C. Data-dependent acquisition (DDA) mode was applied, with a full-scan resolution of 60,000, an *m*/*z* range of 300–1800, and a maximum injection time of 50 ms.

### 2.8. Determination of Minimum Inhibitory Concentration (MIC)

The minimum inhibitory concentration (MIC) of crude antimicrobial lipopeptides against *A. jinshanensis* AJS1 was determined using a broth microdilution method [42]. Crude lipopeptides were dissolved in methanol to create a series of concentration gradients: 6, 12, 24, 48, 96, 192, 384, 768, and 1536 μg/mL. *A. jinshanensis* AJS1 was inoculated into 96-well microtiter plates at a final concentration of 1 × 10^7^ CFU/mL. Each well contained a total volume of 200 μL, consisting of bacterial suspension and lipopeptide solution at the desired concentration. Plates were incubated anaerobically at 30 °C for 48 h, and bacterial growth was assessed by measuring the optical density at 600 nm using BioTek microplate reader (Agilent Technologies, Santa Clara, CA, USA).

### 2.9. Stability Assessment of Crude Lipopeptides

The storage and physicochemical stability of crude lipopeptides were evaluated. For storage stability, crude lipopeptides were stored at 4 °C for 14 days, after which their relative inhibition rate was measured. To assess physicochemical stability, crude lipopeptides were subjected to different conditions, including pH 2–6 for 2 h, temperatures ranging from 30 to 60 °C for 1 h, and ethanol concentrations of 0–5% (*v*/*v*) for 2 h. Untreated samples stored at 4 °C, pH 7, and 0% ethanol were used as controls.

The relative inhibition rate was calculated as shown below, where the blank diameter corresponded to the diameter of the agar punch:

(3) $$Relative\;inhibition\;rate=\frac{\left(inhibition\;zone\;diameter\;of\;treated\;crude\;lipopeptides-blank\;diameter\right)}{\left(inhibition\;zone\;diameter\;of\;control\;crude\;lipopeptides-blank\;diameter\right)}\times 100\%$$

### 2.10. Analysis of the Antimicrobial Mechanism

Propidium iodide (PI) staining and extracellular alkaline phosphatase (AKPase) activity assays were performed with minor modifications based on previously reported methods [15]. Protein and DNA leakage assays were conducted with slight modifications according to the method described by Yi et al. [43]. *A. jinshanensis* AJS1 cell suspensions were treated with crude lipopeptides at a final concentration of 0.5× MIC, while sterile water was used as the negative control. Samples were incubated anaerobically at 30 °C for 5 h.

For PI staining, cells were washed twice with sterile phosphate-buffered saline (PBS) and resuspended in PBS containing 10 μg/mL PI (Sangon Biotech, Shanghai, China). After incubation at room temperature for 30 min in the dark, fluorescence intensity was measured using BioTek microplate reader (Agilent Technologies, Santa Clara, CA, USA) with excitation and emission wavelengths of 536 nm and 617 nm, respectively. For extracellular AKPase activity determination, samples were centrifuged at 10,000 rpm for 10 min at 4 °C to collect the supernatant. AKPase activity was quantified using a commercial alkaline phosphatase assay kit (Sangon Biotech, Shanghai, China) following the manufacturer’s instructions. For protein and DNA leakage assays, 1 μL of culture supernatant was analyzed using a NanoDrop One spectrophotometer (Thermo Fisher Scientific, Waltham, MA, USA) to determine extracellular protein and double-stranded DNA concentrations.

### 2.11. Statistical Analysis

Unless otherwise specified, all experiments in this study were conducted with three biological replicates. The normality of the experimental data was evaluated using the Shapiro–Wilk test, where a *p*-value greater than 0.05 indicates conformity to a normal distribution. For skewed data, the Kruskal–Wallis test was applied.

## 3. Results

### 3.1. Screening of Antagonistic High-Temperature Daqu

**Figure 1:**
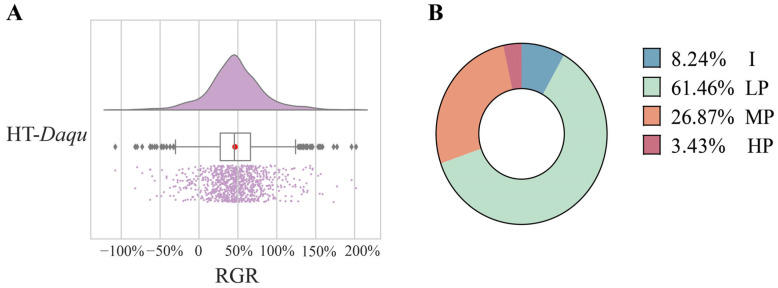
Screening of high-temperature *Daqu*. The inhibition/promotion effects shown apply specifically to *A. jinshanensis* AJS1. (**A**) Relative growth rates of 934 high-temperature *Daqu* samples. (**B**) Proportions of high-temperature *Daqu* exhibiting inhibition (I, <0), low promoting (LP, 0–60%), moderate promoting (MP, 60–120%), and high promoting (HP, >120%).

A total of 934 high-temperature *Daqu* samples were screened, with an average relative growth rate (RGR) of 46.3% (Figure 1A). Overall, 91.76% of the *Daqu* samples promoted the growth of *A. jinshanensis* AJS1 to varying degrees. Among these, low-level promotion accounted for the largest proportion (61.46%), representing the most common effect of high-temperature *Daqu* on *A. jinshanensis* AJS1 growth (Figure 1B).

### 3.2. Microbial Community Characteristics of High-Temperature Daqu with Different Relative Growth Rates

**Figure 2:**
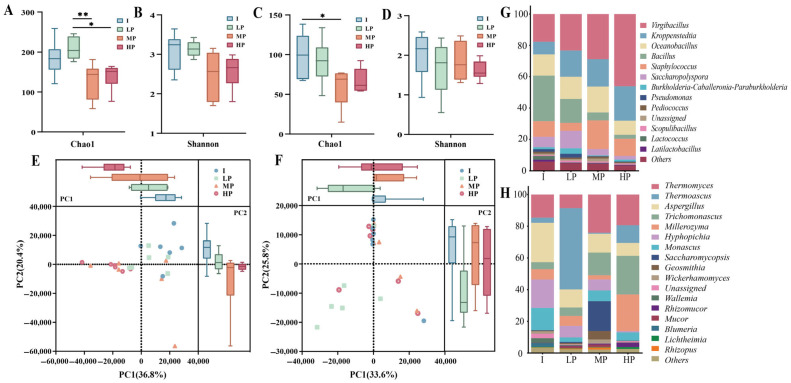
Microbial community structure of high-temperature *Daqu* with different relative growth rates of *A. jinshanensis* AJS1. (**A**,**B**) Chao1 and Shannon indices of bacteria in high-temperature *Daqu* with different relative growth rates. (**C**,**D**) Chao1 and Shannon indices of fungi in high-temperature *Daqu* with different relative growth rates. (**E**,**F**) PCA analysis of bacterial and fungal communities in high-temperature *Daqu* with different relative growth rates. (**G**,**H**) Genus-level analysis of bacteria and fungi in high-temperature *Daqu* with different relative growth rates. * indicates *p* < 0.05, meaning the difference between groups was statistically significant; ** indicates *p* < 0.01, meaning the difference between groups was statistically significant.

A total of 24 high-temperature Daqu samples were analyzed (6 samples per group), with each sample subjected to 16S rRNA and ITS amplicon sequencing once. Significant differences were observed among groups in bacterial α-diversity indices (Chao1 and Shannon) and fungal Chao1 index (ANOVA, *p* < 0.05). The mean values of these indices were higher in the low promoting (LP) and inhibitory (I) Daqu groups than in the moderately promoting (MP) and highly promoting (HP) groups (Figure 2A–C). In contrast, no significant differences were detected in fungal Shannon diversity among the groups (Figure 2D). Principal component analysis (PCA) further revealed differences in microbial community structures among high-temperature Daqu groups with different RGRs. Among them, PC1 of the bacterial and fungal communities explained 36.8% and 33.6% of the variation in the bacterial and fungal communities, respectively. Notably, greater differences in RGR among groups corresponded to larger relative distances along bacterial PC1, indicating a positive correlation between bacterial community dissimilarity and differences in *A. jinshanensis* AJS1 growth responses (Figure 2E).

### 3.3. Taxonomic Composition of Microbial Communities in High-Temperature Daqu

At the genus level, 212 bacterial genera were detected across all samples, among which 13 dominant genera exhibited an average relative abundance exceeding 1% in at least one group. Six genera showed average relative abundances greater than 1% across all groups: *Virgibacillus*, *Kroppenstedtia*, *Oceanobacillus*, *Bacillus*, *Staphylococcus*, and *Saccharopolyspora* (Figure 2G). A total of 141 fungal genera were identified, with 17 genera showing an average relative abundance above 1% in at least one group. Five fungal genera exhibited average relative abundances greater than 1% across all groups: *Thermomyces*, *Aspergillus*, *Trichomonascus*, *Millerozyma*, and *Monascus* (Figure 2H). The distribution of the top 30 bacterial and fungal genera across samples is shown in Figure S1.

### 3.4. Differential Microorganisms Associated with Relative Growth Rate Categories

**Figure 3:**
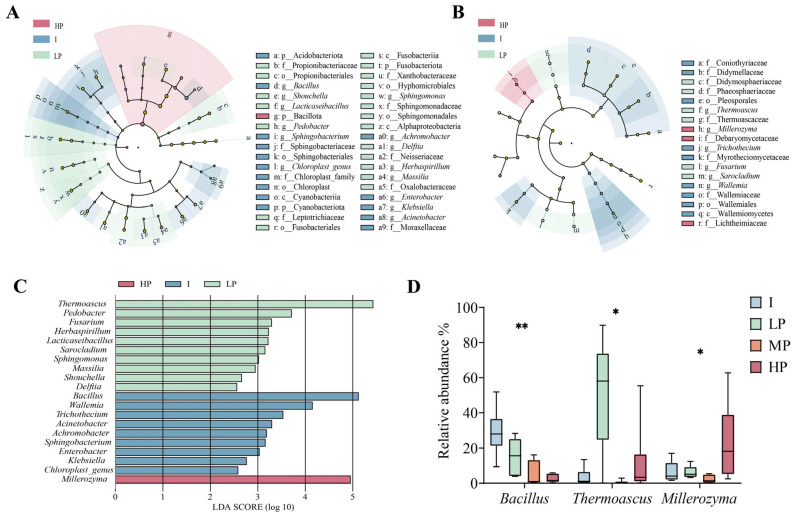
Differential microbes in high-temperature *Daqu* with different relative growth rates. (**A**,**B**) LEfSe analysis of bacteria and fungi in high-temperature *Daqu* with different relative growth rates. (**C**) Significantly enriched bacteria and fungi in high-temperature *Daqu* with different relative growth rates. (**D**) Significantly enriched bacteria and fungi at the genus level with an average relative abundance > 1% in each group in high-temperature *Daqu* with different relative growth rates. * indicates *p* < 0.05, meaning the difference between groups was statistically significant; ** indicates *p* < 0.01, meaning the difference between groups was statistically significant.

The numbers of shared and unique amplicon sequence variants (ASVs) among bacterial and fungal communities in different groups are shown in Figure S2. LEfSe analysis was performed separately for bacterial and fungal datasets (Figure 3A,B) using |LDA| > 2 and *p* < 0.05 as thresholds. This threshold is a widely accepted standard in microbial community differential analysis, effectively balancing sensitivity and specificity. It mitigates the risk of false positive results that can arise from excessively low thresholds, ensuring that the identified differential genera possess both significant biological relevance and statistical significance [44]. A total of 20 significantly differential genera were identified, including 9 enriched in the inhibitory (I) group, 10 in the low promoting (LP) group, and 1 in the highly promoting (HP) group (Figure 3C). By integrating LEfSe results with the top 10 most abundant bacterial and fungal genera, three core differential microorganisms were identified: *Bacillus* (I group), *Thermoascus* (LP group), and *Millerozyma* (HP group) (Figure 3D). Kruskal–Wallis test indicated that all the three aforementioned genera exhibited significant inter-group differences in relative abundance.

### 3.5. Prediction of Microbial Interactions

Spearman correlation analysis was conducted among the top 10 most abundant bacterial and fungal genera. The correlation heatmap revealed a significant positive correlation between *Hyphopichia* and *Bacillus*, whereas *Virgibacillus* and *Kroppenstedtia* exhibited significant negative correlations with *Bacillus*. *Burkholderia-Caballeronia-Paraburkholderia*, *Pseudomonas*, and *Thermomyces* were positively correlated with *Thermoascus*, while *Virgibacillus*, *Staphylococcus*, *Hyphopichia*, *Monascus*, and *Wickerhamomyces* showed significant negative correlations with *Thermoascus*. No genus showed a significant positive or negative correlation with *Millerozyma* (Figure S3). Notably, *Virgibacillus* exhibited significant negative correlations with both *Bacillus* (core differential microorganism in the I group) and *Thermoascus* (core differential microorganism in the LP group). *Hyphopichia* showed a significant positive correlation with *Bacillus* but a significant negative correlation with *Thermoascus*.

### 3.6. Selection of Antagonistic Strains

**Figure 4:**
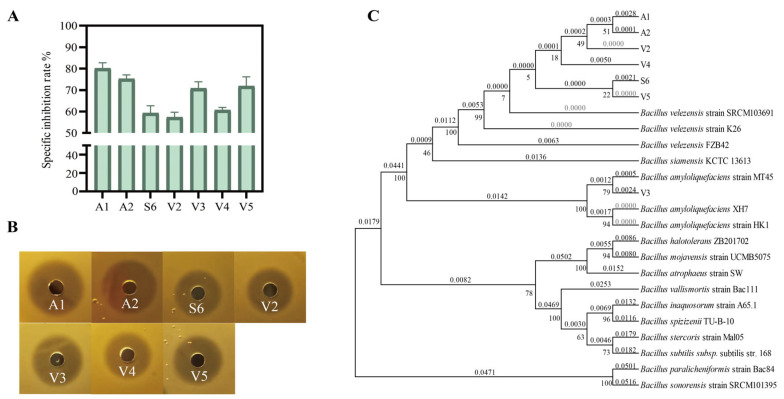
Selection of antagonistic strains against *A. jinshanensis* AJS1. (**A**) Specific inhibition rates of the seven antagonistic strains. (**B**) Inhibition zone of 7 strains culture supernatant against *A. jinshanensis* AJS1. (**C**) Phylogenetic tree of the seven strains based on *gyrA*.

Among the 33 *Bacillus* strains tested, *B*. *cereus* (1 strain), *B*. *licheniformis* (20 strains), and *B*. *sonorensis* (5 strains) showed no antagonistic activity against *A. jinshanensis* AJS1. Seven antagonistic strains against *A. jinshanensis* AJS1 were obtained, with relative inhibition rates ranging from 57% to 80%. Among them, strain V2 exhibited the weakest inhibition (57.3%), whereas strain A1 showed the strongest antagonistic activity, with a relative inhibition rate of 80.3% (Figure 4A). The inhibition zone of 7 strains culture supernatant against *A. jinshanensis* AJS1 were shown in Figure 4B. Except for strain V3, which is *Bacillus amyloliquefaciens*, all the others are *B*. *velezensis* (Figure 4C). Growth and carbon source utilization analyses revealed that all seven strains exhibited a similar growth pattern, characterized by an initial increase in OD_600_ followed by a decline and stabilization. For strain V4, residual glucose content dropped below 10% at 48 h, coinciding with the highest OD_600_. For strain V3, residual glucose fell below 10% at 36 h, with maximum OD_600_ achieved at 24 h. For the remaining strains, residual glucose dropped below 10% and OD_600_ peaked at 24 h (Figure S4). Given its highest relative inhibition rate and favorable growth characteristics, strain A1 was selected for subsequent analyses.

### 3.7. Identification of the Antagonistic Strain

**Figure 5:**
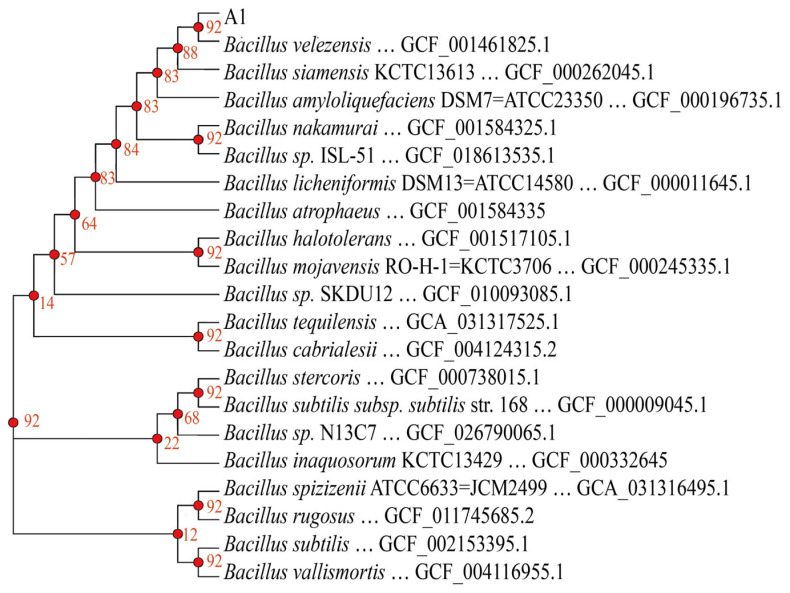
Core-gene phylogenetic tree of *B. velezensis* A1.

Average nucleotide identity (ANI) analysis revealed that strain A1 shared >90% genomic similarity with *B. velezensis* NRRL B-41580, *B. siamensis* KCTC 13613, and *B. amyloliquefaciens* DSM 7. Among these, the highest ANI value (98.6%) was observed with *B. velezensis* NRRL B-41580 (Table S3). Comparative BLAST analysis demonstrated genomic variations between strain A1 and these reference strains (Figure S5). Phylogenetic analysis based on core genes further showed that strain A1 clustered most closely with *B. velezensis* NRRL B-41580 (Figure 5), consistent with the ANI results. Accordingly, strain A1 was identified as *B. velezensis*.

### 3.8. Genome Assembly and Annotation of B. velezensis A1

**Figure 6:**
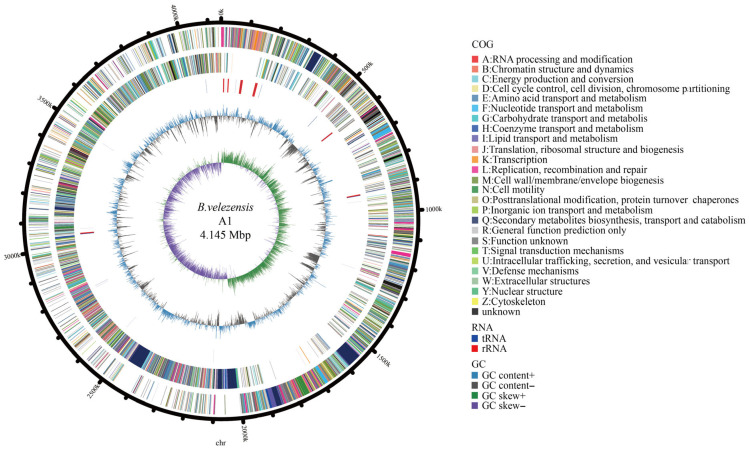
Circular genome map of strain A1 chromosome. From innermost to outermost: the first ring denotes GC skew; the second ring indicates GC content; the third and fourth rings show the positions of tRNA and rRNA on the genome; the fifth and sixth rings display CDS on the plus and minus strands (different colors represent different COG categories of CDS); the outermost ring represents the chromosome karyotype.

Genome assembly of *B. velezensis* A1 yielded four circular replicons with a total genome size of 4.254 Mb. Genome annotation identified 4415 open reading frames (ORFs), accounting for 88.30% of the total genome length. The genome consisted of one circular chromosome and three plasmids, with an average GC content of 45.93% (Figure 6 and Figure S6). A total of 3829 protein-coding genes were assigned to COG functional categories, of which 2859 genes were functionally annotated. Further GO Slim annotation classified 2641 genes into biological process categories, 2600 genes into cellular component categories, and 1497 genes into molecular function categories. Mapping of Kyoto Encyclopedia of Genes and Genomes (KEGG) orthologs revealed that 2202 genes were assigned to KEGG pathways, including 292 genes involved in amino acid metabolism and 86 genes associated with lipid metabolism (Figure S7).

### 3.9. Metabolic System and CAZyme Analysis of B. velezensis A1

Genome annotation revealed that *B. velezensis* A1 harbors a total of 150 carbohydrate-active enzyme (CAZyme) genes, including 3 polysaccharide lyases, 7 auxiliary activity enzymes, 16 carbohydrate-binding modules, 27 carbohydrate esterases, 41 glycosyltransferases, and 56 glycoside hydrolases (GHs). Among these, GH family enzymes represented the largest proportion, accounting for 37.3% of all CAZyme genes (Figure S8).

### 3.10. Secondary Metabolite Biosynthetic Gene Cluster Analysis of B. velezensis A1

Genome mining using antiSMASH identified a total of 12 secondary metabolite biosynthetic gene clusters in the genome of *B. velezensis* A1. Among these, seven clusters showed high similarity (>85%) to previously characterized biosynthetic gene clusters. These seven clusters were annotated as encoding the lipopeptides surfactin and fengycin, the ribosomally synthesized and post-translationally modified peptide bacilysin, the macrolide macrolactin H, the polyketide–macrolactin phosphate ester difficidin, the hybrid polyketide–nonribosomal peptide bacillaene, and the siderophore bacillibactin. In addition, five gene clusters exhibited low similarity (<10%) to known clusters or showed no matches in the antiSMASH database, suggesting the presence of potentially novel secondary metabolites (Figure S9).

### 3.11. Crude Lipopeptide Extraction and MIC Determination

**Figure 7:**
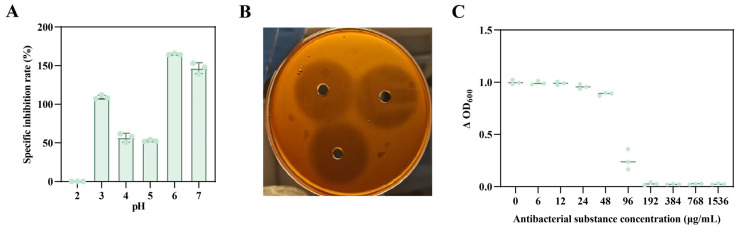
Crude lipopeptides from *B. velezensis* A1 and determination of their minimum inhibitory concentration (MIC). (**A**) Relative inhibition rates of precipitates obtained from *B. velezensis* A1 culture supernatant at different pH values against *A.jinshanensis* AJS1. (**B**) Inhibition zone of the antimicrobial fraction precipitated at pH 6 from *B. velezensis* A1 against *A. jinshanensis* AJS1. (**C**) Minimum inhibitory concentration (MIC) of the crude lipopeptide fraction from *B. velezensis* A1 against *A. jinshanensis* AJS1.

Among the fermentation supernatants of *B. velezensis* A1, the precipitate obtained at pH 6 exhibited the strongest inhibitory activity against *A. jinshanensis* AJS1 (Figure 7A,B). Using a two-step acid precipitation–methanol extraction procedure, crude antimicrobial lipopeptides were successfully obtained. The minimum inhibitory concentration (MIC) of the crude lipopeptides against *A. jinshanensis* AJS1 was determined to be 192 μg/mL (Figure 7C).

### 3.12. Purification and Structural Identification of Antimicrobial Lipopeptides

**Figure 8:**
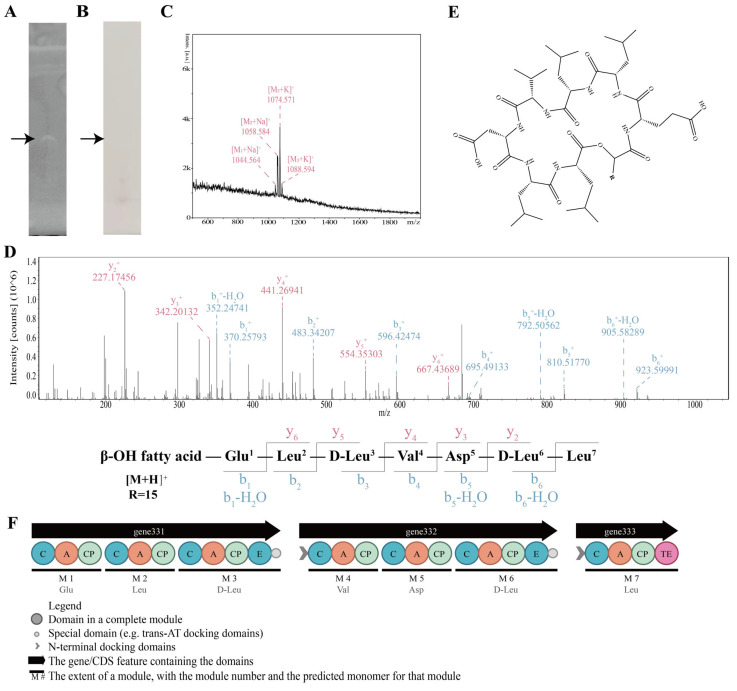
Purification and identification of the antimicrobial lipopeptides produced by *B. velezensis* A1. (**A**) TLC detection of lipids in the antimicrobial fraction from *B. velezensis* A1. (**B**) TLC detection of free amino groups in the antimicrobial fraction from *B. velezensis* A1. (**C**) MALDI-TOF-MS identification of lipopeptide components in the antimicrobial fraction from *B. velezensis* A1. (**D**) LC-MS/MS identification of lipopeptide components in the antimicrobial fraction from *B. velezensis* A1 (exemplified by [M + H]^+^ = 1036.68706). (**E**) Molecular formula of surfactin; R = (CH_2_)_n_, *n* = 12–14. (**F**) Surfactin biosynthetic gene clusters in *B. velezensis* A1. Original maps generated with antiSMASH 7.1.0 and modified by the authors.

Crude lipopeptides were further purified by preparative TLC. After development, a single prominent band was observed, which exhibited both lipid-positive and free amino group–negative staining, indicating the presence of a lipopeptide structure (Figure 8A,B).

MALDI-TOF mass spectrometry analysis of the purified fraction revealed a series of characteristic molecular ion peaks in the m/z range of 1040–1090 (Figure 8C). The mass (M_1_~M_3_) differences between adjacent peaks were approximately 14 Da, consistent with variations in fatty acid chain length. These molecular weight distributions corresponded well to those reported for surfactin-family lipopeptides.

Further structural elucidation was performed using LC-MS/MS. Fragmentation patterns of the precursor ions confirmed the presence of a cyclic peptide backbone linked to a β-hydroxy fatty acid chain. The detected b- and y-ion series matched the conserved amino acid sequence motifs of surfactin. LC-MS/MS analysis further confirmed that these compounds were C14–C16 surfactins. The MS/MS spectrum of C15 surfactin ([M_2_ + H]^+^ = 1036.68706) is shown in Figure 8D, while the spectra of C14 ([M_1_ + H]^+^ = 1022.67186) and C16 ([M_3_ + H]^+^ = 1050.70366) surfactins are provided in Figure S10. The peptide moieties of the purified surfactins were consistent with the structures predicted from genome analysis (Figure 8E). The biosynthetic gene cluster modules responsible for surfactin production are shown in Figure 8F.

### 3.13. Stability of Crude Lipopeptides

**Figure 9:**
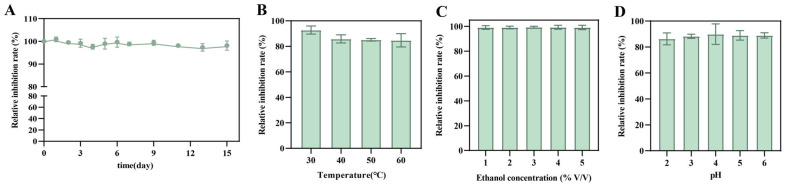
Stability of the crude lipopeptide extract from *B. velezensis* A1. (**A**) Storage stability of the crude lipopeptide extract at 4 °C. (**B**–**D**) Stability of the crude lipopeptide extract under different temperatures, ethanol concentrations, and pH conditions.

The storage and physicochemical stability of the crude lipopeptides were further evaluated. After storage at 4 °C for 14 days, the crude lipopeptides retained a relative inhibition rate of 98.1%, indicating excellent storage stability (Figure 9A). Physicochemical stability was assessed under conditions representative of *Jiang*-flavor *Baijiu* fermentation, including variations in temperature, ethanol concentration, and pH. At 30 °C and 60 °C, the relative inhibition rate of the crude lipopeptides remained above 80%, indicating good thermal stability (Figure 9B). Across ethanol concentrations ranging from 1% to 5% (*v*/*v*), the crude lipopeptides maintained relative inhibition rates exceeding 95% (Figure 9C). Under acidic conditions, the relative inhibition rate of the crude lipopeptides remained above 80% (Figure 9D).

### 3.14. Mechanism of Action of the Crude Lipopeptide

**Figure 10:**
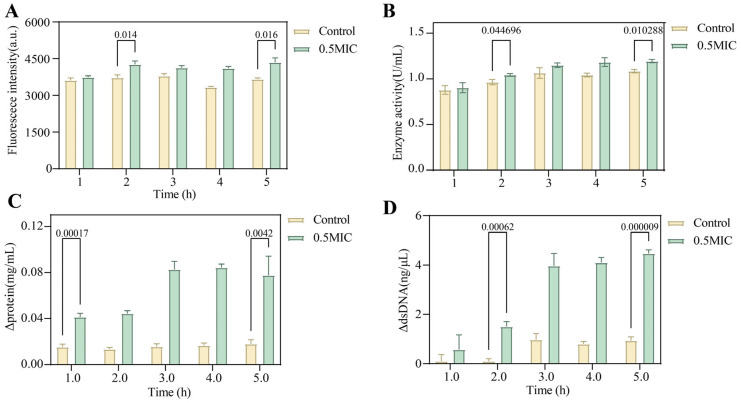
Mechanism of the crude lipopeptide extract from *B. velezensis* A1 against *A. jinshanensis* AJS1. (**A**) PI staining of *A. jinshanensis* AJS1 after treatment with the crude lipopeptide extract from *B. velezensis* A1. (**B**) AKPase activity of *A. jinshanensis* AJS1 after treatment with the crude lipopeptide extract from *B. velezensis* A1. (**C**,**D**) Protein and dsDNA concentrations in the supernatant of *A. jinshanensis* AJS1 after treatment with the crude lipopeptide extract from *B. velezensis* A1.

PI staining, extracellular alkaline phosphatase (AKPase) activity, and extracellular protein and DNA levels were used to evaluate the integrity of the cell wall and membrane. Compared with the control, treatment with the crude lipopeptide significantly increased PI fluorescence intensity in *A. jinshanensis* AJS1 at 2 h and further increased it by 18.8% at 5 h (Figure 10A). The crude lipopeptide also significantly enhanced extracellular AKPase activity at 2 h and increased it by 10.1% at 5 h relative to the control (Figure 10B). Moreover, treatment markedly elevated extracellular protein and dsDNA contents at 1 and 2 h, and their levels increased by 60 μg/mL and 3.5 μg/mL, respectively, at 5 h compared with the control (Figure 10C,D).

## 4. Discussion

Excessive acidity reduces the quality of sauce-flavor *Baijiu*, and *A. jinshanensis* is a major contributor to acidity increase during fermentation [2,5,17]. High-temperature *Daqu* acts as a key reservoir of microorganisms and metabolites, with the potential to provide antagonistic strains and inhibitory compounds [12,18,19]. However, its inhibitory effect on *A. jinshanensis* remains poorly understood. In this study, we compared the microbial community structures of different high-temperature *Daqu* types that exerted distinct effects on the growth of *A. jinshanensis* AJS1, and selected antagonistic strains based on the differential genera. Using whole-genome sequencing, we then predicted the inhibitory metabolites produced by the isolate with the strongest antagonistic activity. Finally, we isolated and characterized the inhibitory compound and evaluated its stability and mode of action, providing new strategies for lactate regulation in *Baijiu* fermentation.

Our results showed significant differences in both bacterial and fungal communities among *Daqu* types with different effects on *A. jinshanensis* AJS1 growth, and the bacterial community exhibited a stronger determinative effect than the fungal community. At the genus level, the dominant genera shared across groups were consistent with previous reports [19,45]. By integrating LEfSe results with the top 10 dominant bacterial and fungal genera, we identified three key taxa: *Bacillus*, *Thermoascus*, and *Millerozyma*. Among them, *Bacillus* displayed the highest relative abundance in inhibitory *Daqu* (I) and was negatively correlated with the relative growth rate of *A. jinshanensis* AJS1. Notably, the ecological inference regarding the role of *Bacillus* in *Daqu* in this study is primarily based on microbial abundance and inhibitory effects, which have not been directly validated through functional experiments. Previous research has established that *Bacillus* is a crucial microbial component in high-temperature *Daqu*, with inoculation of species such as *B. velezensis*, *B. licheniformis*, or *B*. *subtilis* enhancing *Daqu* quality [46,47,48]. In high-temperature *Daqu*, an increase in *Bacillus* abundance significantly alters the expression of various metabolic pathways and transforms community interactions from a cooperative state to a mixed competitive–cooperative interaction state, a shift potentially regulated by *Bacillus*-mediated quorum sensing [19]. Specifically, during the initial stage of *Daqu* fermentation, a notable negative correlation is observed between *Pediococcus* and *Bacillus* [49]. Furthermore, the combined treatment with *Monascus* and *Bacillus* enhances the capacity for flavor regulation [50]. Additionally, previous studies have demonstrated that the concentrations of volatile compounds, such as esters, pyrazines, and alcohols, in *Daqu* significantly increase following inoculation with *B. velezensis* and *Bacillus subtilis* [51]. For practical validation, future research will focus on pilot-scale and industrial-scale fermentation experiments to evaluate ecological interactions and the flavor of *Baijiu* under actual production conditions.

Prior studies have demonstrated that *B. nakamurai* fermentation supernatants inhibit biofilm formation and planktonic growth of *L. fermentum*, *L. plantarum*, and *L. brevis* [52]; that *B. licheniformis* BL-4 secretes bacteriocins that inhibit *L. panis* via cell membrane and wall disruption [15]; and that mixed starters containing *B. subtilis* DB821 reduce viable counts of *Enterococcus faecium* and *E. faecalis* [53]. Although antagonism of *Bacillus* species against *A. jinshanensis* has not been reported, the observed community differences and prior knowledge suggest that *Bacillus* possesses the potential to antagonize *A. jinshanensis*.

**Figure 11:**
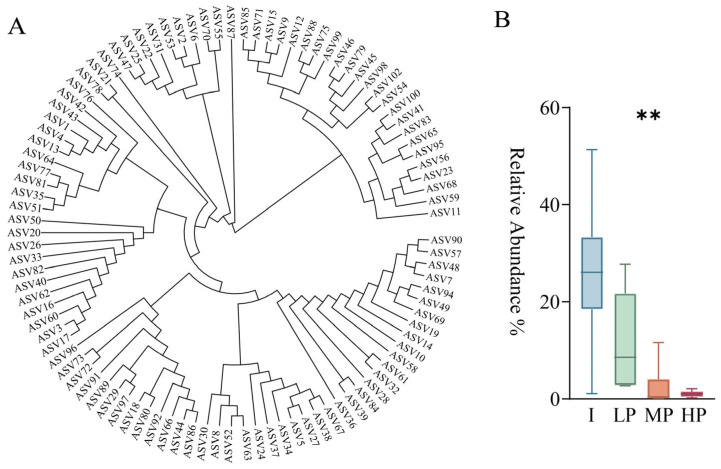
Phylogenetic tree based on high-abundance bacteria ASVs and distribution of *B. velezensis* and *B. amyloliquefaciens* in each *Daqu* group. (**A**) Phylogenetic tree using the representative sequences of the 100 most abundant bacteria ASVs. (**B**) Combined abundance of *B. velezensis* and *B. amyloliquefaciens* in each *Daqu* group (I, inhibition; LP, low promoting; MP, moderate promoting; HP, high promoting). ** indicates *p* < 0.01, meaning the difference between groups was statistically significant.

Selection of *Bacillus* strains isolated from high-temperature *Daqu* showed that *B. velezensis* and *B. amyloliquefaciens* exerted antagonistic activity against *A. jinshanensis* AJS1, whereas *B*. *licheniformis*, *B*. *sonorensis*, and *B*. *cereus* did not. Among these five *Bacillus* species, the surfactin biosynthetic gene cluster was detected mainly in the genomes of *B. amyloliquefaciens* and *B. velezensis* [54], whereas the lichenysin cluster was found in *B. licheniformis* and *B. sonorensis* [55]. The primary structural difference between surfactin (Glu^1^) and lichenysin (Gln^1^) lies in the first amino acid residue. Previous studies have reported that surfactin exhibits stronger antagonistic activity than lichenysin against certain Gram-positive bacteria [56,57] and is generally produced at higher levels in wild-type strains [58], which may explain the observed inter-species differences in antagonism. A phylogenetic tree was constructed with QIIME2 using the representative sequences of the 100 most abundant bacteria ASVs. *Bacillus*-derived sequences among them were selected for BLAST analysis using the NCBI database. Results showed ASVs 3, 16, 17, 60, and 62 were annotated as either *B. velezensis* or *B. amyloliquefaciens*. In the phylogenetic tree, these five ASVs clustered closely together (Figure 11A), consistent with their close evolutionary relationship to *B. velezensis* and *B. amyloliquefaciens* [28]. Their combined abundance in each *Daqu* group (I, LP, MP, HP) is shown in Figure 11B. Kruskal–Wallis test indicated that there are significant inter-group differences in relative abundance.

Among all strains selected, *B. velezensis* A1 showed the strongest inhibitory effect on *A. jinshanensis* AJS1. Whole-genome annotation showed that A1 possesses sporulation capability and diverse primary and secondary metabolic pathways, reflecting high ecological adaptability and nutritional versatility consistent with previous studies on *B. velezensis* [59,60,61].

Based on the experiments, our results demonstrated that *B. velezensis* A1 antagonizes *A. jinshanensis* AJS1 through C14–C16 surfactins, whose amino-acid composition (Glu^1^-Leu^2^-(D-)Leu^3^-Val^4^-Asp^5^-(D-)Leu^6^-Leu^7^) matched the genomic predictions. Moreover, our findings experimentally validated a previous ANI-based conclusion that the surfactin A (Leu^7^) lineage is present in *B. velezensis* [62]. Although no studies have reported the MIC of surfactin against lactic acid bacteria, previous research has demonstrated that surfactin exhibits antibacterial activity against various *Lactobacillus* species, including *Lactobacillus fermentum*, *Lactobacillus johnsonii*, *Lactobacillus amylovorus*, and *Lactobacillus brevis* [63]. Furthermore, surfactin has been utilized to mitigate food spoilage caused by *Lactobacillus* [64]. Additionally, the MIC values of surfactin against Gram-positive bacteria, such as *Micrococcus flavus*, *Mycobacterium smegmatis*, and *Bacillus cereus*, range from 50 to 200 μg/mL [65], which is comparable to the MIC value of 192 μg/mL of crude surfactin against *A. jinshanensis* AJS1 obtained in this study. The evolution of resistance to cyclic lipopeptides may be constrained or delayed due to the universal nature of their mechanism of action, which targets biofilm structures [66]. Experiments involving *Staphylococcus aureus* have confirmed that surfactin does not readily induce in vitro resistance [67].

Whole-genome sequencing revealed that *B*. *velezensis* A1 harbors 12 secondary metabolite biosynthetic gene clusters. While this study confirmed that surfactin is the primary active compound against *A*. *jinshanensis* AJS1, the existing literature suggests that these secondary metabolites may exert a synergistic antimicrobial effect [61]. For instance, bacilysin exerts an antibacterial effect by inhibiting cell wall synthesis, and its combination with surfactin, which disrupts cell membranes, may establish a dual-target antimicrobial mechanism that enhances inhibitory activity [54]. The synergistic effects of these metabolites on *A. jinshanensis* warrant further verification through in vitro combination experiments in future studies.

During sauce-flavor *Baijiu* fermentation, the environment is acidic, high-temperature, and contains ethanol [5,11]. We found that the crude lipopeptide exhibited strong stability (relative inhibition rate > 80%) at pH 2–6 and 30–60 °C, consistent with earlier reports [68], and maintained excellent stability (relative inhibition rate > 95%) at ethanol concentrations of 1–5%. This provides a mechanistic basis for the observed increase in activity under alkaline conditions.

We further elucidated the mechanism by which the crude lipopeptide antagonizes *A. jinshanensis* AJS1. PI cannot penetrate intact membranes, entering only membrane-damaged cells [69,70]. The significant increase in PI fluorescence after treatment indicates membrane disruption by the lipopeptide. AKPase, located between the cell wall and membrane, increases in activity when the cell wall is damaged or intracellular components leak [71,72]. Although AKPase activity increased after treatment, the magnitude (10.1% at 5 h) was lower than the increase in PI fluorescence (18.8%), suggesting that the lipopeptide may target the membrane rather than directly degrading the cell wall, leading to leakage of periplasmic and membrane-associated AKPase. Measurements of extracellular DNA and protein further confirmed significant increases after treatment, indicating membrane disruption and release of intracellular contents [73,74]. Compared to traditional physical acid reduction methods or process-improved acid reduction, the introduction of *Bacillus* strains isolated from *Daqu*, along with their metabolites, for acidity control during *Baijiu* fermentation is advantageous for preserving the original flavor while also reducing investment costs [15]. Antibacterial lipopeptides exhibit several beneficial properties, including low molecular weight, good thermal stability, excellent water solubility, non-immunogenicity, non-toxicity, and minimal side effects [75]. Surfactin has been utilized in the preservation of various food products and is generally recognized as both safe and effective [64]. Despite its general acceptance as safe, there has yet to be a comprehensive evaluation of the safety levels of surfactin. Future studies should conduct a thorough safety evaluation that encompasses the application range and dosage of *Bacillus*-derived lipopeptides.

It is important to acknowledge that this study is limited to in vitro culture experiments and has not yet explored the ecological behavior of *B. velezensis* A1 within the solid matrix of high-temperature *Daqu* and fermented grains. The production of lipopeptide families is significantly influenced by the bacterial strain used, along with the nutritional and environmental conditions. Previous studies have demonstrated that the *B. velezensis* GA1 and FZB42 strains exhibit higher surfactin production under solid-state fermentation conditions compared to submerged fermentation. Conversely, the *B. velezensis* S499 strain shows the opposite trend [76]. Therefore, subsequent studies will further investigate the metabolic profiling of *B. velezensis* A1 under solid-state fermentation conditions. In terms of the key yeasts during the fermentation process, surfactins generally exhibit antibacterial and antiviral activity, whereas fungal membranes containing ergosterol show low sensitivity or resistance to surfactin [75,77]. This observation suggests that the surfactins produced by *B. velezensis* A1 may have a limited impact on yeasts, thereby supporting their potential application in fermentation. This will require further validation through co-cultivation experiments under solid-state fermentation conditions in future research. In conclusion, this study identified microorganisms and metabolites within high-temperature *Daqu* that antagonize *A. jinshanensis* AJS1. *Bacillus* was the most differential genus between inhibitory (I) and promoting (LP, MP, HP) *Daqu* types and was a dominant genus in high-temperature *Daqu*. We characterized the genome of *B. velezensis* A1—the most effective antagonistic strain—and isolated surfactin homologs whose structures matched genomic predictions. These surfactins antagonized *A. jinshanensis* AJS1 by damaging the cell membrane and causing cytoplasmic leakage. These findings provide theoretical support for biological acid-reduction strategies in sauce-flavor *Baijiu* fermentation. For the practical application of this biological acid-reduction strategy, several aspects warrant further investigation: validation at pilot and industrial scales within actual fermentation processes, a cost-benefit analysis, and an evaluation of economic feasibility, as well as the exploration of synergistic effects with other functional microorganisms. These future studies will facilitate the translation of our findings from laboratory research to industrial application.

## Data Availability

The original contributions presented in this study are included in the article and Supplementary Materials. Further inquiries can be directed to the corresponding authors.

## Supplementary Materials

The following supporting information can be downloaded at: https://www.mdpi.com/article/10.3390/foods15071140/s1, Figure S1: Genus-level bubble plots of microbial communities in high-temperature *Daqu* with different relative growth rates. (A) Bacterial genus-level bubble plot. (B) Fungal genus-level bubble plot. Figure S2: Numbers of unique and shared ASVs in high-temperature *Daqu* with different relative growth rates. (A) Numbers of unique and shared bacterial ASVs. (B) Numbers of unique and shared fungal ASVs. Figure S3: Correlation heatmap of the top 10 bacterial and fungal genera by relative abundance (unclassified genera excluded) in high-temperature *Daqu*. Figure S4: Growth and carbon-source consumption of seven *Acetilactobacillus jinshanensis*-antagonistic strains in Landy medium. (A) OD_600_ increase. (B) Glucose consumption. Figure S5: BLAST Ring Image Generator (BRIG) comparison of *Bacillus velezensis* A1 with type strains of *B. velezensis*, *B. siamensis*, and *B. amyloliquefaciens*. From outermost to innermost: *B. amyloliquefaciens* type strain, *B. siamensis* type strain, *B. velezensis* type strain, and strain A1. Figure S6: Functional maps of the *Bacillus velezensis* A1 plasmids. (A–C) Circos maps of plasmids 1–3 of strain A1. From innermost to outermost: ring 1, GC skew; ring 2, GC content; rings 3–4, tRNA and rRNA positions; rings 5–6, CDS on the plus and minus strands (different colors indicate different COG categories); outermost ring, plasmid karyotype. Figure S7: Genome annotation of *Bacillus velezensis* A1. (A–C) General gene annotation and classification of *B. velezensis* A1 based on COG, GO, and KEGG databases. Figure S8: CAZy functional classification of *Bacillus velezensis* A1. Figure S9: Secondary-metabolite prediction for *Bacillus velezensis* A1. Original map generated with antiSMASH 7.1.0 and modified by the authors. Figure S10: MS/MS spectra of C14 and C16 surfactins. (A) C14 surfactin. (B) C16 surfactin. Table S1: *Bacillus* strains used in this study. Table S2: 92 conserved single-copy core genes. Table S3. ANI values between strain A1 and 20 closely related species.

## Abbreviations

The following abbreviations are used in this manuscript:

RGR	Relative growth rate
I	Inhibitory
LP	Low promoting
MP	Moderately promoting
HP	Highly promoting
ASV	Amplicon sequence variant
ANI	Average nucleotide identity
TLC	Thin-layer chromatography
DDA	Data-dependent acquisition
MIC	Minimum inhibitory concentration
AKPase	Alkaline phosphatase
PBS	Phosphate-buffered saline
PCA	Principal component analysis
PI	Propidium iodide
LC-MS/MS	Liquid Chromatography-Tandem Mass Spectrometry
MALDI-TOF	Matrix-Assisted Laser Desorption/Ionization Time-of-Flight
KEGG	Kyoto Encyclopedia of Genes and Genomes
CDS	Coding Sequence
COG	Clusters of Orthologous Groups
CAZyme	Carbohydrate-Active Enzyme
GH	glycoside hydrolase
ITS	Internal Transcribed Spacer
pKa	negative logarithm of the acid dissociation constant (negative log of Ka)
BLAST	Basic Local Alignment Search Tool
NR	Non-Redundant (database)
GO	Gene Ontology
BRIG	BLAST Ring Image Generator
ANOVA	Analysis of Variance
LEfSe	Linear Discriminant Analysis Effect Size
UBCG	Up-to-date Bacterial Core Gene
