# Supplementary material for: Surfactin-Producing Bacillus velezensis A1 Inhibits Lactic Acid Bacteria in Jiang-Flavor Baijiu Fermentation
Source: Foods. 2026 Mar 26;15(7):1140. doi: 10.3390/foods15071140 (PMC13073612; doi:10.3390/foods15071140)
Supplement: Supplementary file 1 [file foods-15-01140-s001.zip › foods-4180233-supplementary.pdf]

foods

MDPI

## Supplementary Figures

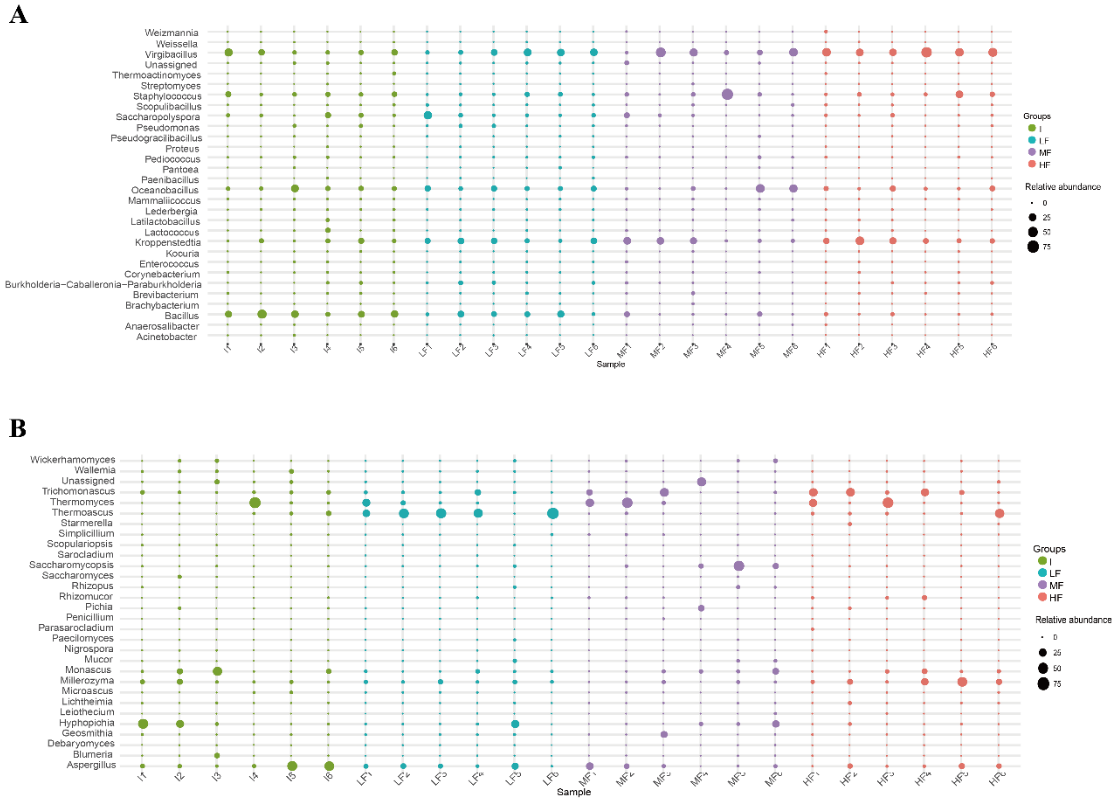


**Figure S1.** Genus-level bubble plots of microbial communities in high-temperature *Daqu* with different relative growth rates. (A) Bacterial genus-level bubble plot. (B) Fungal genus-level bubble plot.

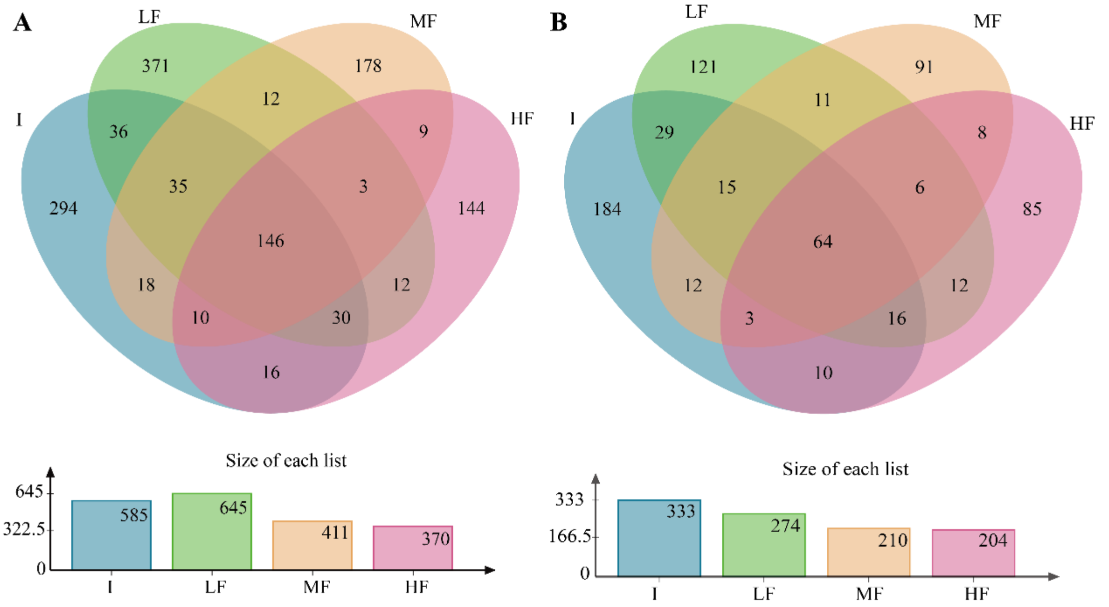


**Figure S2.** Numbers of unique and shared ASVs in high-temperature *Daqu* with different relative growth rates. (A) Numbers of unique and shared bacterial ASVs. (B) Numbers of unique and shared fungal ASVs.

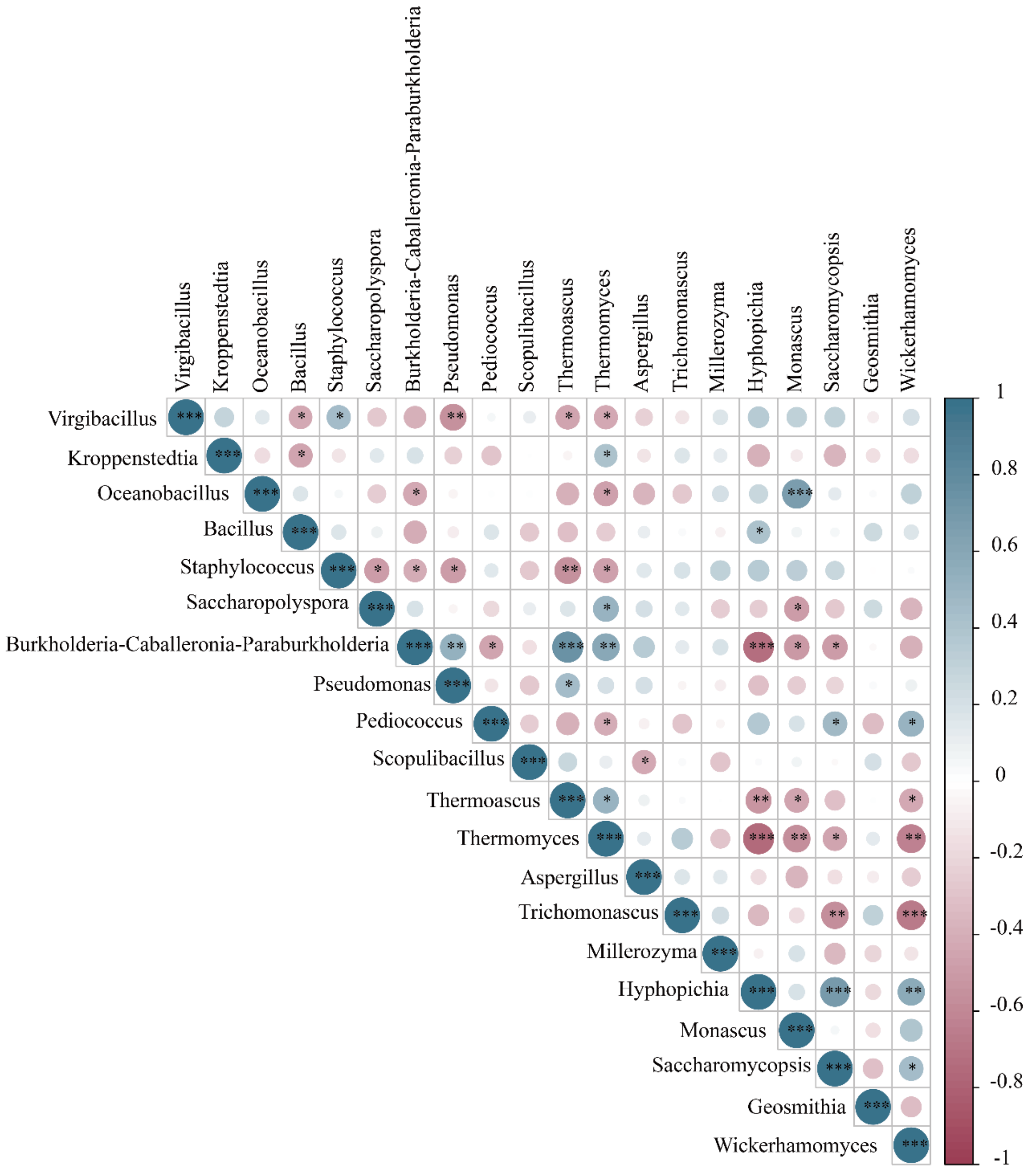


**Figure S3.** Correlation heatmap of the top 10 bacterial and fungal genera by relative abundance (unclassified genera excluded) in high-temperature *Daqu*.

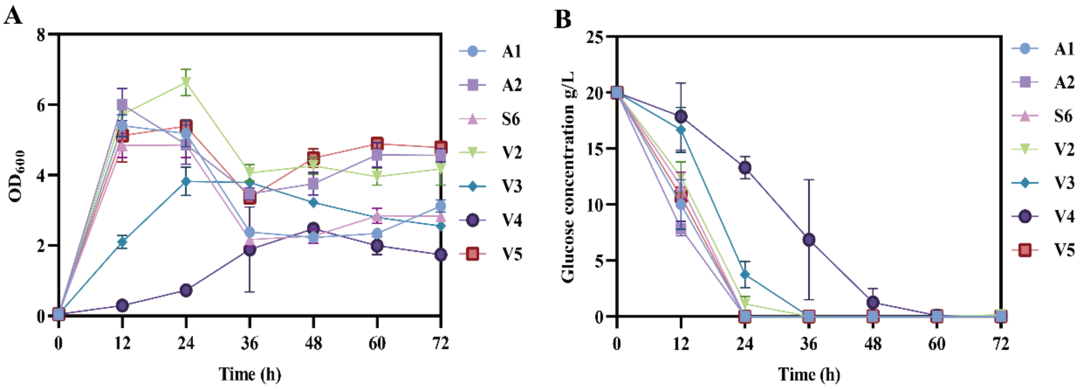


**Figure S4.** Growth and carbon-source consumption of seven *Acetilactobacillus jinshanensis*-antagonistic strains in Landy medium. (A) OD600 increase. (B) Glucose consumption.

https://doi.org/10.3390/foods15071140

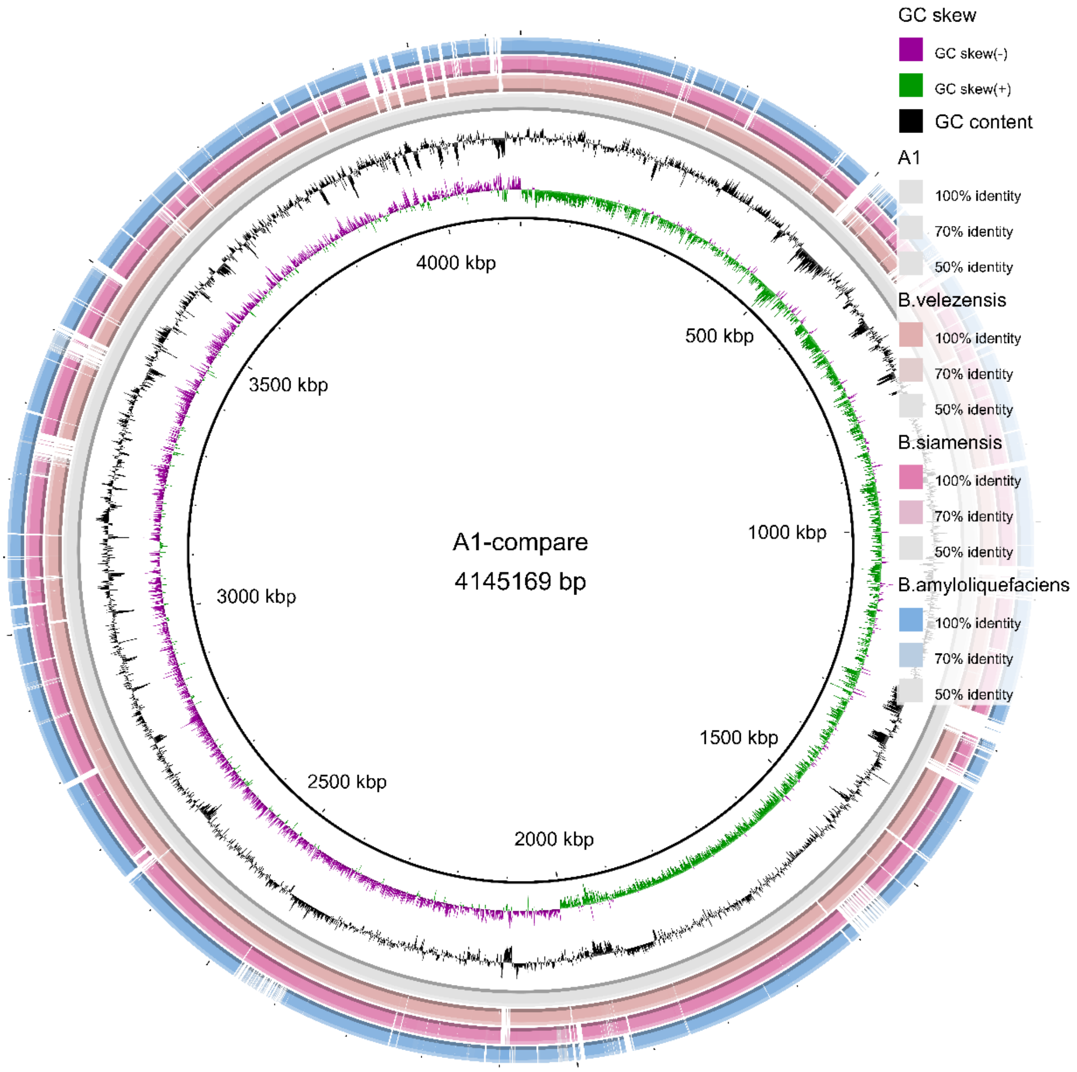


**Figure S5.** BLAST Ring Image Generator (BRIG) comparison of *Bacillus velezensis* A1 with type strains of *B. velezensis*, *B. siamensis*, and *B. amyloliquefaciens*. From outermost to innermost: *B. amyloliquefaciens* type strain, *B. siamensis* type strain, *B. velezensis* type strain, and strain A1.

https://doi.org/10.3390/foods15071140

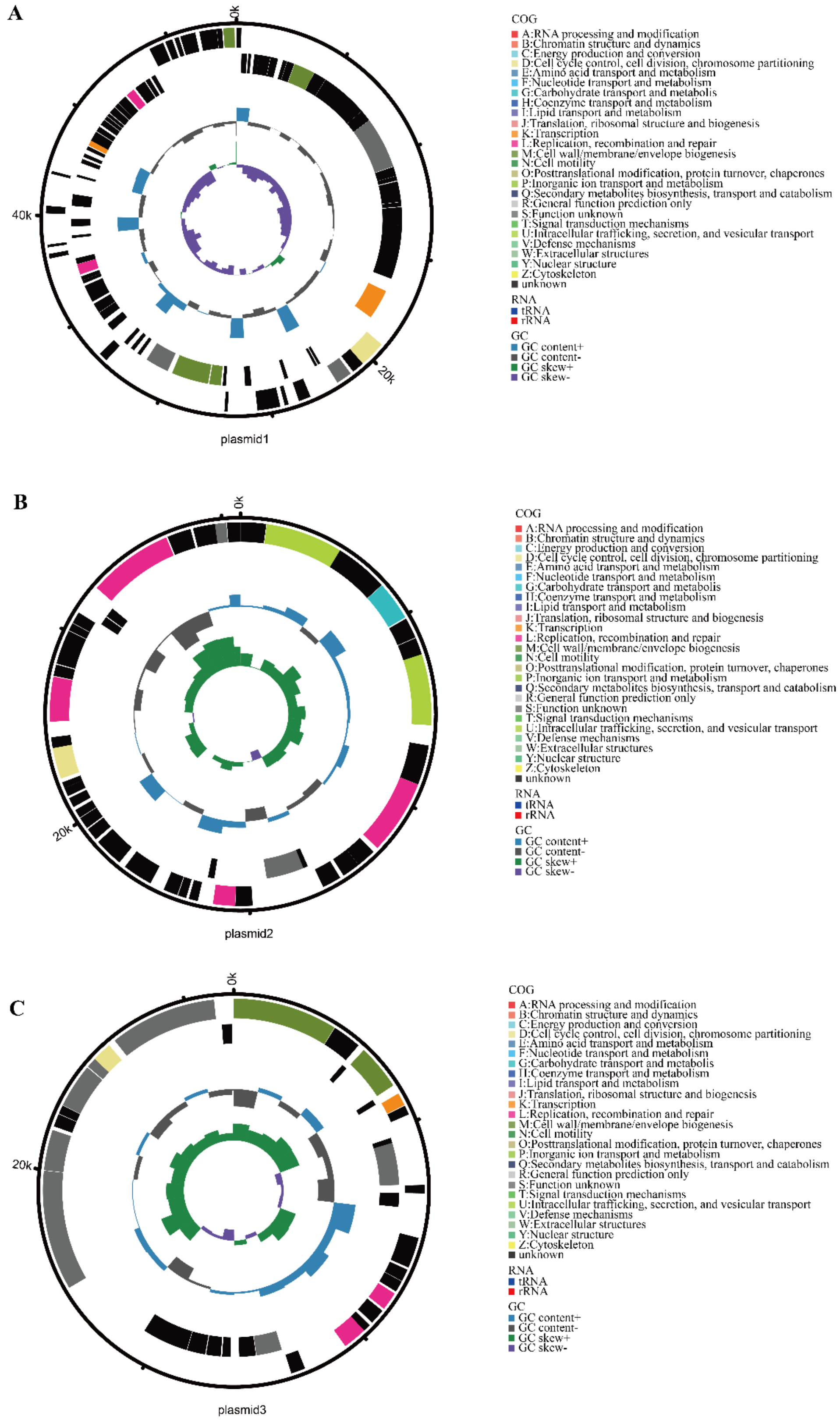


**Figure S6.** Functional maps of the *Bacillus velezensis* A1 plasmids. (A–C) Circos maps of plasmids 1–3 of strain A1. From innermost to outermost: ring 1, GC skew; ring 2, GC content; rings 3–4, tRNA and rRNA positions; rings 5–6, CDS on the plus and minus strands (different colors indicate different COG categories); outermost ring, plasmid karyotype.

https://doi.org/10.3390/foods15071140

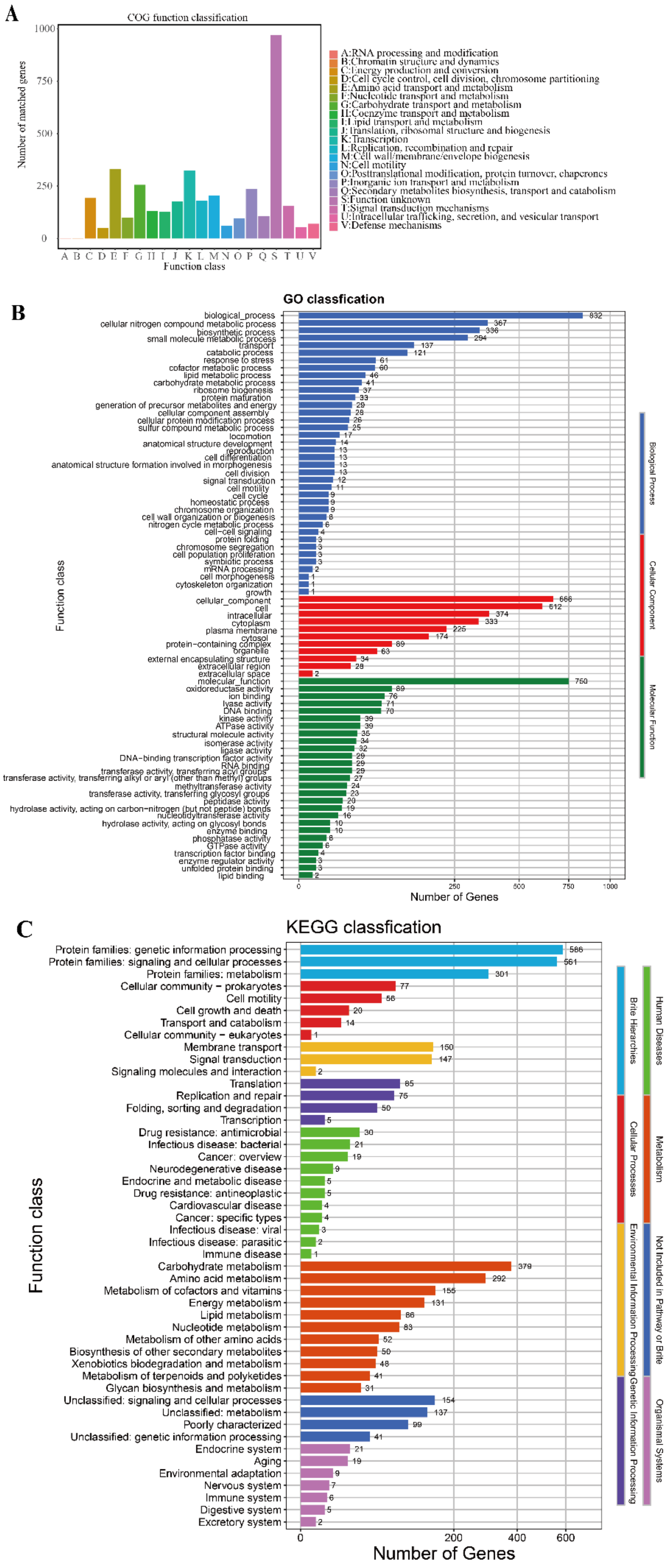


**Figure S7.** Genome annotation of *Bacillus velezensis* A1. (A–C) General gene annotation and classification of *B. velezensis* A1 based on COG, GO, and KEGG databases.

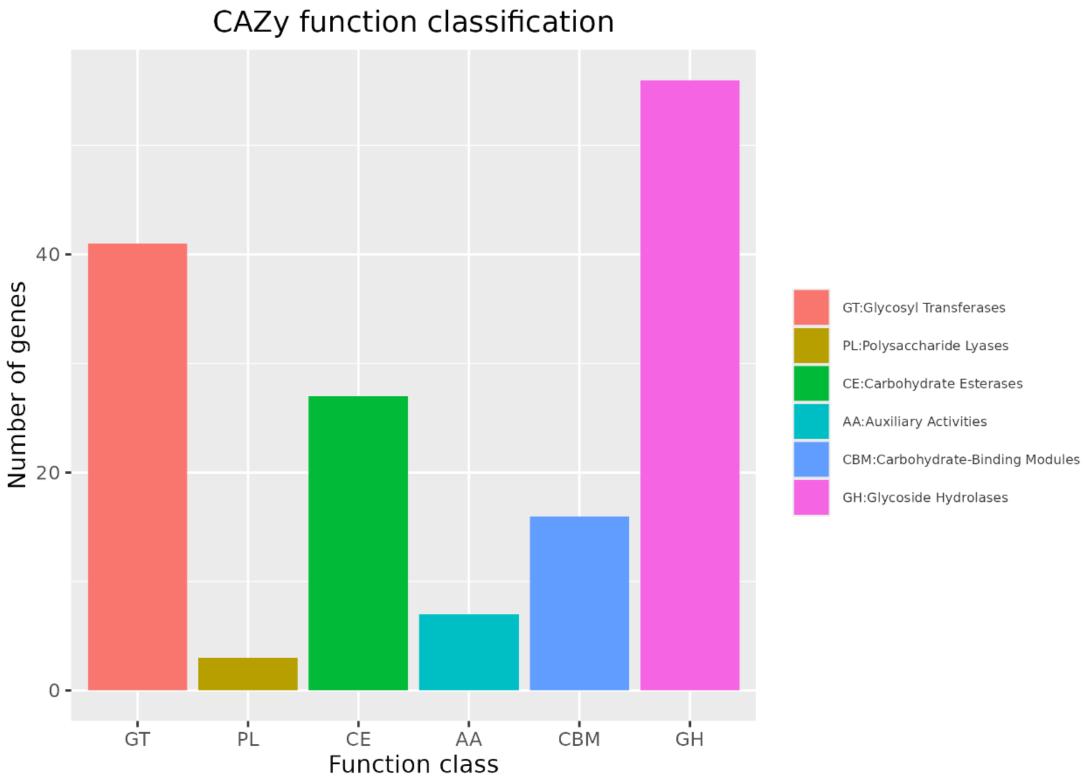


**Figure S8.** CAZy functional classification of *Bacillus velezensis* A1.

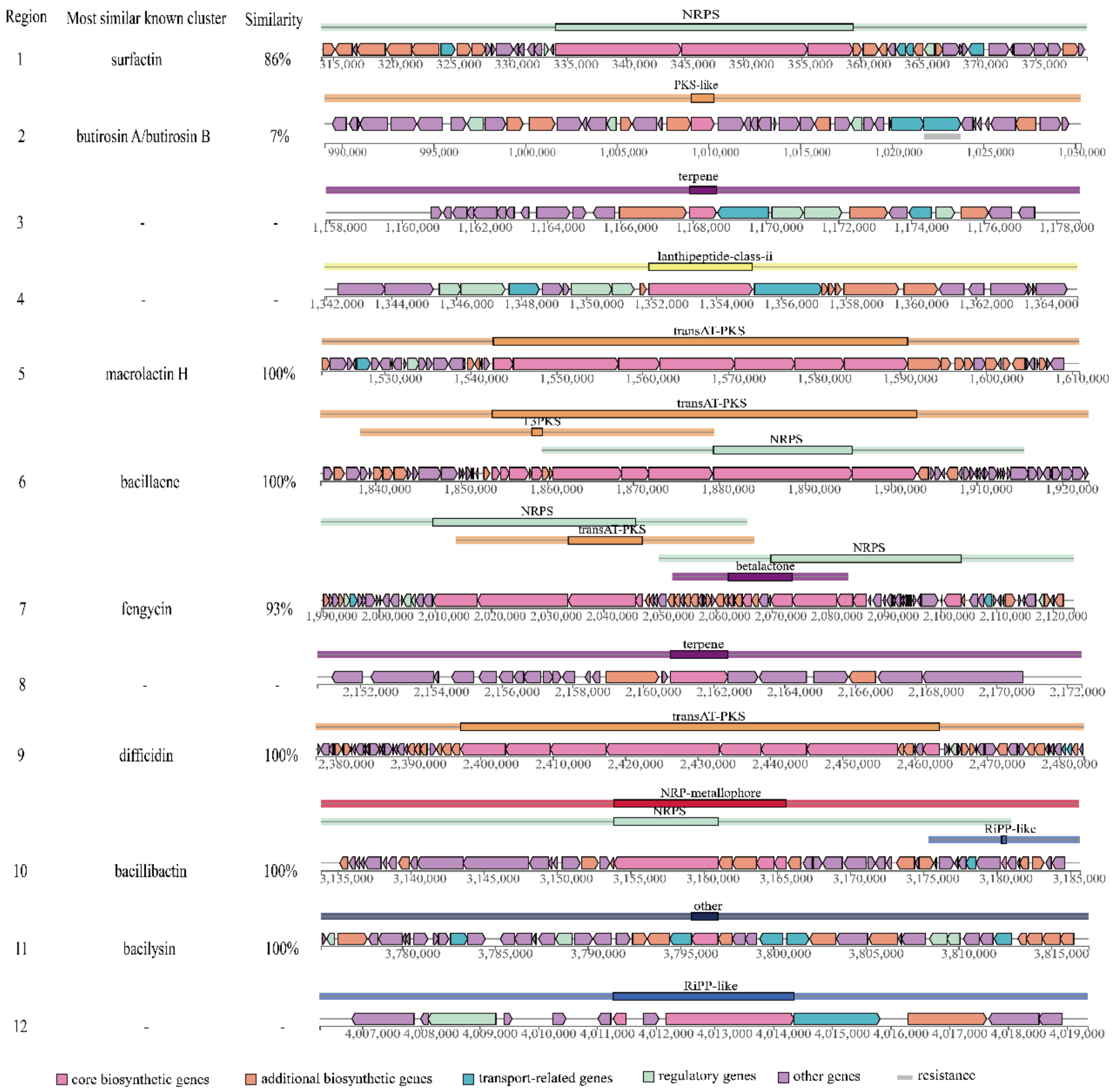


**Figure S9.** Secondary-metabolite prediction for *Bacillus velezensis* A1. Original map generated with antiSMASH 7.1.0 and modified by the authors.

https://doi.org/10.3390/foods15071140

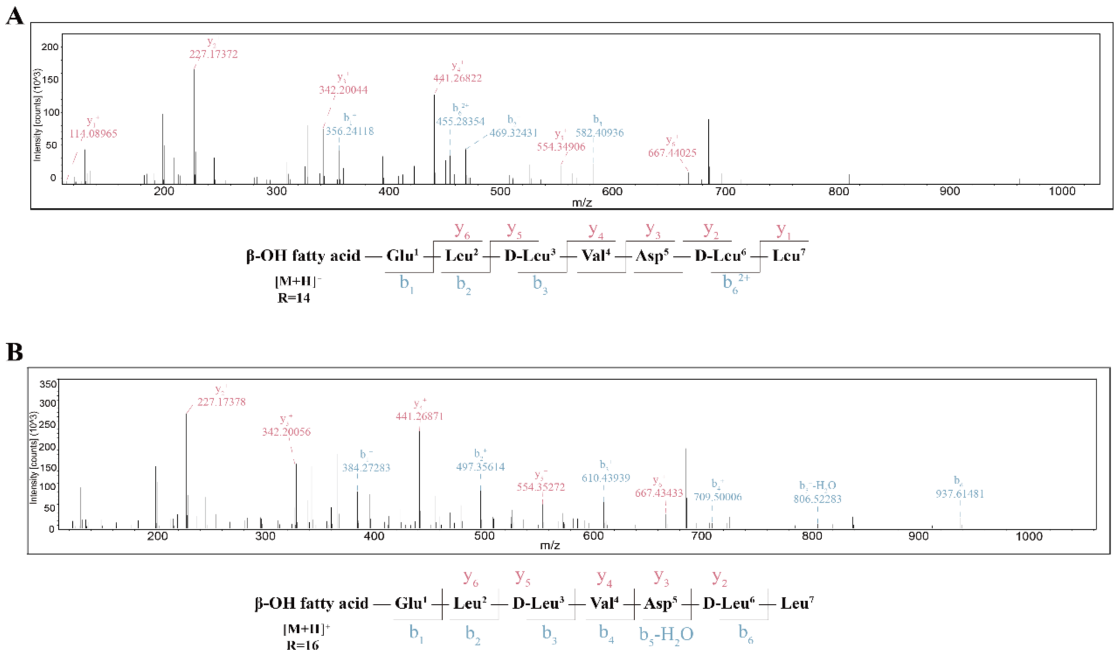


**Figure S10.** MS/MS spectra of C14 and C16 surfactins. (A) C14 surfactin. (B) C16 surfactin.

■ **Supplementary Tables**

**Table S1.** *Bacillus* strains used in this study.

| Strain designation in this study | Corresponding designation in the original isolation literature | Species | Gene sequencing-based identification |
|---|---|---|---|
| L1 | BL-1 | *Bacillus licheniformis* | 16S rRNA |
| L2 | BL-2 | *Bacillus licheniformis* | 16S rRNA |
| L3 | BL-3 | *Bacillus licheniformis* | 16S rRNA |
| L4 | BL-4 | *Bacillus licheniformis* | 16S rRNA |
| L5 | BL-5 | *Bacillus licheniformis* | 16S rRNA |
| L6 | BL-6 | *Bacillus licheniformis* | 16S rRNA |
| L7 | BL-7 | *Bacillus licheniformis* | 16S rRNA |
| L8 | BL-8 | *Bacillus licheniformis* | 16S rRNA |
| L9 | BL-9 | *Bacillus licheniformis* | 16S rRNA |
| L10 | BL-10 | *Bacillus licheniformis* | 16S rRNA |
| L11 | BL-11 | *Bacillus licheniformis* | 16S rRNA |
| L12 | BL-12 | *Bacillus licheniformis* | 16S rRNA |
| L13 | BL-13 | *Bacillus licheniformis* | 16S rRNA |
| L14 | BL-14 | *Bacillus licheniformis* | 16S rRNA |
| L15 | BL-15 | *Bacillus licheniformis* | 16S rRNA |
| L16 | BL-16 | *Bacillus licheniformis* | 16S rRNA |
| L17 | BL-17 | *Bacillus licheniformis* | 16S rRNA |
| L18 | BL-18 | *Bacillus licheniformis* | 16S rRNA |
| L19 | BL-19 | *Bacillus licheniformis* | 16S rRNA |
| L20 | BL-20 | *Bacillus licheniformis* | 16S rRNA |
| S1 | BS-1 | *Bacillus sonorensis* | 16S rRNA |
| S2 | BS-2 | *Bacillus sonorensis* | 16S rRNA |
| S3 | BS-3 | *Bacillus sonorensis* | 16S rRNA |
| S4 | BS-4 | *Bacillus sonorensis* | 16S rRNA |
| S5 | BS-5 | *Bacillus sonorensis* | 16S rRNA |
| BC | BC | *Bacillus cereus* | 16S rRNA |
| A1 | BA-1 | *Bacillus velezensis* | Whole genome (in this study) |
| A2 | BA-2 | *Bacillus velezensis* | gyrA (in this study) |
| S6 | BS-6 | *Bacillus velezensis* | gyrA (in this study) |
| V2 | BV-2 | *Bacillus velezensis* | gyrA (in this study) |
| V4 | BV-4 | *Bacillus velezensis* | gyrA (in this study) |
| V5 | BV-5 | *Bacillus velezensis* | gyrA (in this study) |
| V3 | BV-3 | *Bacillus amyloliquefacien* | gyrA (in this study) |

**Table S2.** 92 conserved single-copy core genes.

| number | gene | number | gene | number | gene | number | gene |
|---|---|---|---|---|---|---|---|
| 1 | alaS | 24 | nusA | 47 | rplP | 70 | rpsJ |
| 2 | argS | 25 | nusG | 48 | rplQ | 71 | rpsK |
| 3 | aspS | 26 | pgk | 49 | rplR | 72 | rpsL |
| 4 | cgtA | 27 | pheS | 50 | rplS | 73 | rpsM |
| 5 | coaE | 28 | pheT | 51 | rplT | 74 | rpsO |
| 6 | cysS | 29 | prfA | 52 | rplU | 75 | rpsP |
| 7 | dnaA | 30 | pyrG | 53 | rplV | 76 | rpsQ |
| 8 | dnaG | 31 | recA | 54 | rplW | 77 | rpsR |
| 9 | dnaX | 32 | rbfA | 55 | rplX | 78 | rpsS |
| 10 | engA | 33 | rnc | 56 | rpmA | 79 | rpsT |
| 11 | ffh | 34 | rplA | 57 | rpmC | 80 | secA |
| 12 | fmt | 35 | rplB | 58 | rpmI | 81 | secG |
| 13 | frr | 36 | rplC | 59 | rpoA | 82 | secY |
| 14 | ftsY | 37 | rplD | 60 | rpoB | 83 | serS |
| 15 | gmk | 38 | rplE | 61 | rpoC | 84 | smpB |
| 16 | hisS | 39 | rplF | 62 | rpsB | 85 | tig |
| 17 | ileS | 40 | rplI | 63 | rpsC | 86 | tilS |
| 18 | infB | 41 | rplJ | 64 | rpsD | 87 | truB |
| 19 | infC | 42 | rplK | 65 | rpsE | 88 | tsaD |
| 20 | ksgA | 43 | rplL | 66 | rpsF | 89 | tsf |
| 21 | lepA | 44 | rplM | 67 | rpsG | 90 | uvrB |
| 22 | leuS | 45 | rplN | 68 | rpsH | 91 | ybeY |
| 23 | ligA | 46 | rplO | 69 | rpsI | 92 | ychF |

https://doi.org/10.3390/foods15071140

**Table S3.** ANI values between strain A1 and 20 closely related species.

| Ref | Species | ANI |
|---|---|---|
| GCF_001461825.1 | Bacillus velezensis | 98.6243 |
| GCF_000262045.1 | Bacillus siamensis KCTC 13613 | 94.1677 |
| GCF_000196735.1 | Bacillus amyloliquefaciens DSM 7 = ATCC 23350 | 93.9701 |
| GCF_018613535.1 | Bacillus sp. ISL-51 | 86.7703 |
| GCF_001584325.1 | Bacillus nakamurai | 86.644 |
| GCF_004124315.2 | Bacillus cabrialesii | 81.2407 |
| GCA_031317525.1 | Bacillus tequilensis | 81.2235 |
| GCF_000009045.1 | Bacillus subtilis subsp. subtilis str. 168 | 81.1671 |
| GCF_000738015.1 | Bacillus stercoris | 81.1501 |
| GCF_000332645.1 | Bacillus inaquosorum KCTC 13429 | 80.9361 |
| GCF_000245335.1 | Bacillus mojavensis RO-H-1 = KCTC 3706 | 80.9045 |
| GCF_011745685.2 | Bacillus rugosus | 80.8871 |
| GCF_004116955.1 | Bacillus vallismortis | 80.8695 |
| GCF_002153395.1 | Bacillus subtilis | 80.816 |
| GCF_026790065.1 | Bacillus sp. N13C7 | 80.807 |
| GCF_001517105.1 | Bacillus halotolerans | 80.7764 |
| GCF_010093085.1 | Bacillus sp. SKDU12 | 80.7367 |
| GCA_031316495.1 | Bacillus spizizenii ATCC 6633 = JCM 2499 | 80.6854 |
| GCF_001584335.1 | Bacillus atrophaeus | 80.5484 |
| GCF_000011645.1 | Bacillus licheniformis DSM 13 = ATCC 14580 | 79.0775 |

https://doi.org/10.3390/foods15071140
